# A fusion-deletion genomic-event underlies poor prognosis in young patients with luminal breast cancer

**DOI:** 10.1371/journal.pone.0349410

**Published:** 2026-06-16

**Authors:** Le-Wei Zheng, Yi-Ming Liu, Ke-Da Yu, Cui-Cui Liu

**Affiliations:** 1 Shanghai Institute of Infectious Disease and Biosecurity, Shanghai Cancer Center and Cancer Institute, Fudan University, Shanghai, China; 2 Department of Breast Surgery, Key Laboratory of Breast Cancer in Shanghai, Cancer Institute, Fudan University Shanghai Cancer Center, Department of Oncology, Shanghai Medical College, Fudan University, Shanghai, China; Fujian Provincial Hospital, CHINA

## Abstract

Breast cancer in young patients, particularly those with the luminal subtype, exhibits more aggressive behavior and poorer clinical outcomes compared with older patients. However, the genomic mechanisms underlying this age-associated aggressiveness remains poorly understood. In this study, we integrated multi-omics data, including gene fusions, mutations, and copy number variations, to investigate age-related molecular heterogeneity in breast cancer. We identified the co-occurrence of the *EEF1AKNMT::DNM3* (*ED*) fusion and *KDM6B* deletion as a novel prognostic biomarker associated with aggressive tumor biology and reduced survival in luminal breast cancer. Moreover, we discovered a widespread pattern of genomic remodeling in early-onset disease, characterized by an increased fusion burden, distinct mutational profiles, and dysregulated transcriptional programs that collectively contribute to high-risk clinical phenotypes. These findings provide mechanistic insights into the enhanced aggressiveness of breast cancer in young patients and identify potential biomarkers for improved risk stratification.

## 1 Introduction

Breast cancer (BC) is one of the most common malignancies among women worldwide and continues to pose a major threat to female health [1–3]. Patients diagnosed at a younger age, often referred to as early-onset BC, tend to experience poorer prognoses than older patients [4], a trend particularly evident in the luminal subtype [5]. Notably, patients under 35 years of age have markedly worse clinical outcomes, with a five-year disease-free survival rate of 77% compared with 91.5% in older patients, even with ovarian function suppression [6]. This unfavorable prognosis in early-onset cases may be closely linked to molecular-level genomic alterations, including gene mutations, copy number variations (CNVs), and fusion genes (FGs). However, detailed analyses stratified by BC subtype remain limited [7,8]. A comprehensive genomic investigation is, therefore, needed to better elucidate the relationship between patient age and BC biology.

FGs are chimeric genes formed through the fusion of two distinct genes, leading to the generation of novel proteins or disruption of normal gene regulation, both of which can have profound biological effects [9]. Although genomic and transcriptomic profiling studies have advanced precision medicine [10–12], investigations into the prognostic relevance of FGs in BC remain scarce. Molecular classification based on genomic, transcriptomic, and proteomic subtypes, combined with gene expression profiling, has enhanced diagnostic accuracy and facilitated individualized treatment [10,13,14]. Moreover, elements of mutational and transcriptomic data have been incorporated into recent molecular classification systems, underscoring the growing importance of precise molecular subtyping [11,13].

A systematic investigation of age-specific FG landscapes is crucial to determine their role in driving the aggressive phenotypes observed in young patients with luminal BC. Tumor gene expression subgroups exhibit substantial age-related heterogeneity across multiple cancers, including leukemia, often driven by recurrent FGs [15]. Furthermore, treatment-related gene mutations can worsen prognosis [16,17]. These molecular and clinical observations provide a framework for understanding the poor outcomes in young patients with luminal BC. However, the molecular mechanisms underlying these differences, particularly the age-specific distribution and prognostic relevance of genetic alterations such as FGs and mutations, remain poorly defined. Few studies have characterized luminal BC from the perspective of FGs, leaving the contribution of age-related FG patterns to poor prognosis an unresolved question.

In this study, we investigated the age-specific distribution of gene fusions by analyzing large-scale cohort data from our center. We hypothesized that gene fusions display age-dependent patterns that contribute to the poor prognosis observed in early-onset BC. We further examined whether specific combinations of FGs and CNVs are associated with more aggressive disease. Finally, we validated the presence of the *EEF1AKNMT::DNM3* fusion (*ED* fusion), which enhances proliferation and migration in estrogen receptor (ER)-positive BC cells. The identification of age-associated molecular markers, including novel FGs, may provide valuable insights for the early detection and stratification of high-risk individuals.

## 2 Materials and methods

### 2.1 Patients and samples

This study utilized data from the FUSCC-BRCA data were available in the Genome Sequence Archive (GSA) database under accession codes PRJCA017539 (https://ngdc.cncb.ac.cn/bioproject/browse/PRJCA017539), a publicly available, de-identified dataset. The original establishment, data collection, and participant consent procedures for the FUSCC dataset were approved by the Medical Ethics Committee of Fudan University Affiliated Cancer Hospital under approval number (ID: 050432–4-2307E). All methods were performed in accordance with the Declaration of Helsinki. We analyzed data from the FUSCC-BRCA cohort to identify novel FGs, explore co-occurring genomic alterations, and evaluate their associations with clinical outcomes. The FUSCC-BRCA cohort is a multi-omics dataset comprising 351 Chinese patients with BC who underwent treatment at the Department of Breast Surgery, Fudan University Shanghai Cancer Center (FUSCC), between September 2009 and October 2015. Eligible participants met the following inclusion criteria: (1) female patients diagnosed with unilateral invasive breast carcinoma; (2) pathological confirmation of tumor specimens by the Department of Pathology at FUSCC, with estrogen receptor (ER), progesterone receptor (PR), and human epidermal growth factor receptor 2 (HER2) status independently verified by two senior pathologists using immunohistochemistry and in situ hybridization; and (3) availability of sufficient frozen tumor tissue for molecular analysis. ER and PR positivity were defined according to American Society of Clinical Oncology (ASCO)/College of American Pathologists (CAP) guidelines as ≥1% positively stained tumor cells. Exclusion criteria included carcinoma in situ, inflammatory BC, and de novo stage IV disease. Three primary clinical endpoints were assessed: distant metastasis-free survival (DMFS), recurrence-free survival (RFS), and overall survival (OS). DMFS was defined as the time from surgery to the occurrence of distant metastasis or death from any cause. RFS was measured from surgery to the first local, regional, or distant recurrence, or death from any cause. OS was defined as the time from surgery to death from any cause. Patients without recorded events were censored at the date of their last follow-up visit.

### 2.2 Analysis of RNA-seq data

Functional enrichment analysis was performed to identify biological pathways and molecular interactions associated with changes in gene expression. Gene Ontology terms and Kyoto Encyclopedia of Genes and Genomes pathways were evaluated using the clusterProfiler package in R (v4.1.1), with gene annotation supported by the org.Hs.e.g.,db database. Statistical significance was set at *p* < 0.05. For FG detection, RNA sequencing reads were first aligned to the GRCh38 reference genome (CTAT_GENOME_LIB, GENCODE v37 build) using the STAR aligner. Putative fusion transcripts were then identified with the STAR-Fusion computational pipeline, which integrates junction-spanning and spanning-read evidence to improve detection accuracy.

### 2.3 Genomic DNA, RNA extraction, and polymerase chain reaction (PCR) assays

Genomic DNA was extracted from BC tissue samples using the TIANamp Genomic DNA Kit (TIANGEN Biotech, Beijing, China), following the manufacturer’s instructions. Total RNA was isolated from cultured cell samples using TRIzol reagent (Invitrogen, Thermo Fisher Scientific Inc., Waltham, MA, USA) according to the manufacturer's standard protocol. RNA concentration and purity were measured using a NanoDrop spectrophotometer (Thermo Fisher Scientific Inc.).

To detect chimeric RNA transcripts, a one-step reverse transcription (RT)-PCR assay targeting specific fusion junctions was performed using the PrimeSTAR® Max DNA Polymerase Kit (Takara Bio Inc., Kusatsu, Japan). The 50 µL reaction mixture consisted of 25 µL of 2 × Platinum SuperFi RT-PCR Master Mix, 2.5 µL each of forward and reverse primers (10 µM), 0.5 µL of reverse transcriptase mix, the RNA template (adjusted to the desired volume according to its concentration), and nuclease-free water to a total volume of 50 µL. Thermal cycling conditions were as follows: initial denaturation at 95 °C for 2 minutes, followed by 40 cycles of 95 °C for 15 seconds, 60 °C for 1 minute, and 72 °C for 1 minute. Primer sequences used for the detection of the *ED* fusion are as follows:

*ED* Forward: GCTCTGTTCCCACTGCTTCATTTGACTACACT;

*ED* Reverse: ACAATGCCCGACCCTCGAGGGAGAAAGTCC.

### 2.4 Gel electrophoresis

Amplified PCR products were analyzed by agarose gel electrophoresis. For each reaction, 10 µL of PCR product was loaded onto a 2% agarose gel prepared in 1 × Tris-borate-EDTA (TBE) buffer and electrophoresed at 140 V for 35 minutes. A 50 bp DNA ladder (10 µL per lane) was loaded at both ends of the gel as a molecular weight reference. Following electrophoresis, DNA bands were visualized and documented using a Gel Doc imaging system (Bio-Rad Laboratories, Inc., Hercules, CA, USA).

### 2.5 Purification of PCR products and sanger sequencing

After electrophoresis, target DNA bands were excised from the agarose gel under ultra violet illumination and purified using the FastPure Gel DNA Extraction Mini Kit (Vazyme, Nanjing, China) according to the manufacturer’s instructions. Purified DNA was eluted in GDP buffer and incubated at 50 °C for 10 minutes to enhance dissolution. DNA concentration and purity were determined using a NanoDrop spectrophotometer (Thermo Fisher Scientific Inc.). Amplicons meeting quality requirements were subjected to bidirectional Sanger sequencing (Sangon Biotech, Shanghai, China) for sequence verification.

### 2.6 Cell viability assay

Cell viability was assessed using the CCK-8 assay (Vazyme, China) according to the standard protocol. Cells were plated in 96-well plates at a density of 3 × 10³ cells/well and cultured for five days. At designated time points, cells were gently washed with phosphate-buffered saline before adding 10 μL of CCK-8 solution per well. Following 2-hour incubation at 37°C, optical density was measured at 450 nm using a microplate reader (BioTek).

### 2.7 Colony formation assay

For colony formation analysis, cells were plated at low density (500 cells/well) in 6-well plates and cultured in BEBM medium supplemented with 1% fetal bovine serum. Following 14 days of incubation, cells were fixed with 4% paraformaldehyde and stained with 0.5% crystal violet solution. Visible colonies (defined as >50 cells) were manually counted, and the colony formation efficiency was calculated as: (number of colonies formed/number of cells seeded) × 100%.

### 2.8 Transwell migration assay

Cells were seeded in serum-free medium into the upper chamber of a transwell insert. After incubation, migrated cells on the lower membrane surface were fixed, stained, and quantified microscopically.

### 2.9 Xenograft tumor model

Four-week-old female BALB/c nude mice, supplied by HFK Bioscience Co., Ltd. (China), were used to establish a xenograft tumor model. The mice were maintained under specific pathogen-free (SPF) conditions with regulated temperature and humidity in the animal facility of Fudan University Shanghai Cancer Center. All experimental protocols involving animals were approved by the Institutional Animal Care and Use Committee of Fudan University (FUSCC‑IACUC‑2025818) and were conducted in accordance with the guidelines issued by the National Academy of Sciences.

The mice were randomly allocated into two groups (n = 5 per group) and each mouse was subcutaneously inoculated with 1 × 10⁶ MCF‑7 cells. Tumor dimensions were measured every nine days. Animals were euthanized either 45 days post‑inoculation or earlier if they displayed signs of pain or distress, such as a hunched posture, reduced mobility, weight loss exceeding 20%, decreased spontaneous activity, loss of appetite, or labored breathing. Euthanasia was performed using carbon dioxide inhalation in compliance with the approved animal protocol, with the aim of alleviating suffering.

Throughout the study, animal health and behavior were monitored daily, and body weight was recorded twice per week. Following euthanasia, tumors were excised, photographed, and weighed for further analysis. Two researchers underwent professional training in animal care or operation and passed the qualification examination.

### 2.10 Quantification and statistical analysis

Statistical analyses were performed using appropriate tests based on variable type. Categorical variables were analyzed using Pearson’s chi-square test or Fisher’s exact test, whereas continuous variables were evaluated using Student’s t-test or the Wilcoxon rank-sum test. Survival analysis was conducted using the survminer package in R (version 4.1.1), and Kaplan–Meier curves were generated to visualize survival outcomes. Multivariate Cox proportional hazards models for OS were constructed using the ezcox package. Age-associated gene expression patterns were evaluated using Pearson’s correlation analysis. Most statistical computations were performed in R (version 4.1.1). Statistical significance was defined as p < 0.05. The meanings of asterisks numbers were *p < 0.05, **p < 0.01.

## 3 Results

### 3.1 Age-related FG patterns predict distinct outcomes in luminal BC

**Fig 1:**
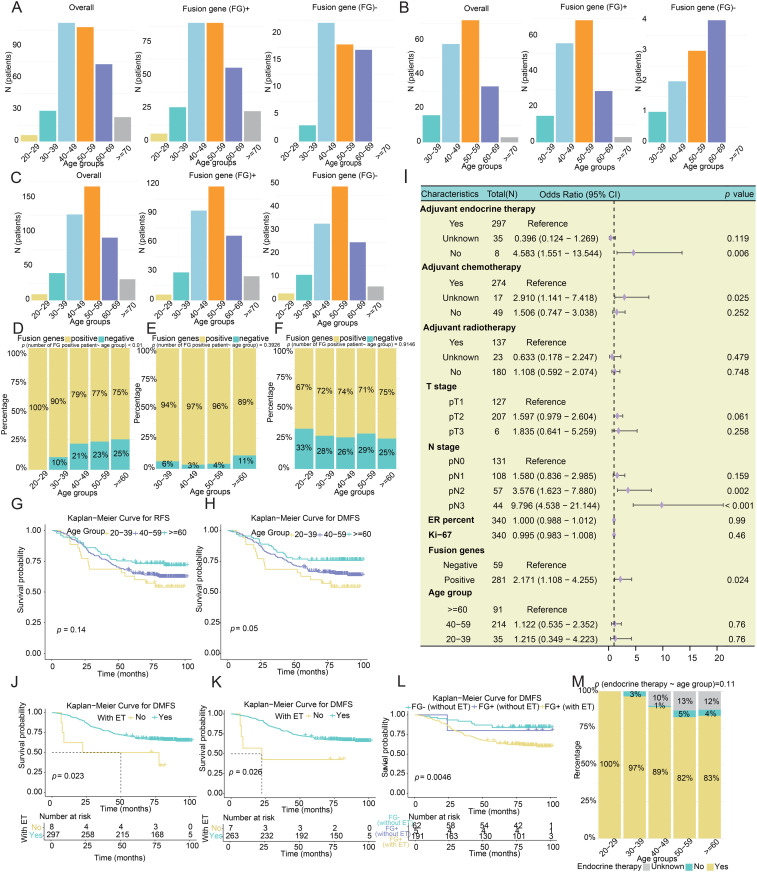
Distribution and prognostic stratification of age groups in distinct subtype and prognostic significance in FUSCC-BRCA cohort. Histograms showed population distribution of the overall, FG+ and FG- subgroup respectively in (*A*) luminal subtype, (*B*)HER2-enriched subtype, and (*C*) TNBC subtype. (D-F) Comparation of the number of FGs between FG+ and FG- patients with BC. (*D*) Percentage bar graphs showed the classes of FG numbers in FG+ (yellow)/ FG- (cyan) in luminal BC. (E) Percentage bar graphs showed the classes of FG numbers in HER2-enriched subtype. (F) Percentage bar graphs showed the classes of FG numbers in TNBC. (G-H) RFS and DMFS analysis (KM curve) of luminal BC patients based on age groups. Comparison of survival curves across the predefined age groups was performed using the log-rank test. (I) Multivariate Cox regression analysis established FG-positive status as a factor with independent prognostic significance. (J) The DMFS prognosis in luminal BC regarding whether to receive endocrine therapy. (K) The DMFS of patients over 40 years old who received endocrine therapy or not. (L) The DMFS for patients over 40 years old based on whether they received ET, further divided into subgroups according to the presence or absence of FGs. (M) The distribution of patients receiving ET among different age groups. FG, fusion gene. BC, breast cancer. TNBC, triple negative breast cancer. RFS, recurrence-free survival. DMFS, distant metastasis-free survival.

A total of 1499 fusion events were identified in luminal BC, 2030 in HER2-enriched tumors, and 2342 in triple-negative BC (TNBC), as detected using the STAR-Fusion tool. We first examined the age distribution patterns across BC subtypes. The incidence of luminal BC peaked in the 40–59-year age group (Fig 1A). Within this group, patients with fusion gene–positive (FG+) luminal tumors tended to be younger, whereas fusion gene–negative (FG–) patients were more prevalent among older age groups, in both FUSCC-BRCA and TCGA cohort (Figs 1A and S1A). We next evaluated the distribution of fusion events in HER2-enriched and TNBC subtypes. In the HER2-enriched subtype, the FG+ group showed no clear age-related trend (Figs 1B and S1B), and a similar absence of age stratification was observed in TNBC (Figs 1C and S1C). To further clarify the relationship between age and FG status, we analyzed the percentage distribution across age groups (Figs 1D, F and S1D). A significant negative correlation was observed between FG+ frequency and increasing age in luminal BC (*p* < 0.01), consistent with prior observations (Fig 1D). No comparable trend was detected in the HER2-enriched or TNBC cohorts (Figs 1E, F and S1D).

Age-stratified survival analyses demonstrated distinct prognostic differences for RFS and DMFS (Figs 1G, H and S1E) Patients aged ≥60 years exhibited the most favorable median outcomes for both recurrence and metastasis (Figs 1G, H and S1F). In multivariate models incorporating clinical covariates, FG+ status emerged as an independent prognostic factor in luminal BC (Fig 1I), whereas this association was not statistically significant in the HER2-enriched or TNBC subtypes. These evidences uncovered that FG+ events are associated with younger age and serve as an independent prognostic factor in luminal BC, whereas this association is not significant in HER2-enriched or TNBC subtypes.

Among hormone receptor–positive (HR^+^) patients, those receiving endocrine therapy (ET+) displayed improved DMFS, RFS, and OS, which is consistent with previous reports (Figs 1J, S2A, B). However, it remains unclear whether FG+ luminal tumors derive additional benefit from endocrine therapy. In patients older than 40 years, ET status continued to significantly affect prognosis (Figs 1K, and S2C-S2D Fig), whereas in those younger than 40 years, no significant survival differences were observed, regardless of ET treatment (S2E-G Fig). Notably, FG+ cases demonstrated early recurrence despite endocrine therapy, and the proportion of patients receiving ET decreased with advancing age (Fig 1L, M). Clinicopathological comparison revealed that FG+ tumors were associated with higher histological grade, greater tumor burden, elevated Ki-67 proliferation index, and lower ER expression compared with FG– tumors (Table 1). Collectively, these findings indicate that FG+ events define a distinct molecular subtype within ER^+^ BC, characterized by intrinsic resistance to conventional endocrine therapy and unfavorable clinical outcomes.

**Table 1:** Baseline characteristics of FG+ and FG- samples.

Characteristics	FG+	FG-	*p* value
n	275	76	
Age, median (IQR)	53 (44, 60)	55 (46, 61)	0.266
Grade, n (%)			< 0.001
1	3 (0.9%)	1 (0.3%)	
2	137 (39%)	56 (16%)	
3	135 (38.5%)	19 (5.4%)	
pT, n (%)			0.009
pT1	92 (26.2%)	39 (11.1%)	
pT2	176 (50.1%)	37 (10.5%)	
pT3	7 (2%)	0 (0%)	
pN, n (%)			0.118
pN0	105 (29.9%)	29 (8.3%)	
pN1	80 (22.8%)	30 (8.5%)	
pN2	46 (13.1%)	12 (3.4%)	
pN3	44 (12.5%)	5 (1.4%)	
Ki67, median (IQR)	30 (20, 40)	20 (15, 30)	< 0.001
ER percent, median (IQR)	90 (85, 90)	90 (85, 95)	0.018

### 3.2 Age-dependent genomic remodeling: Decreasing fusion events and evolving mutational patterns in luminal BC

**Fig 2:**
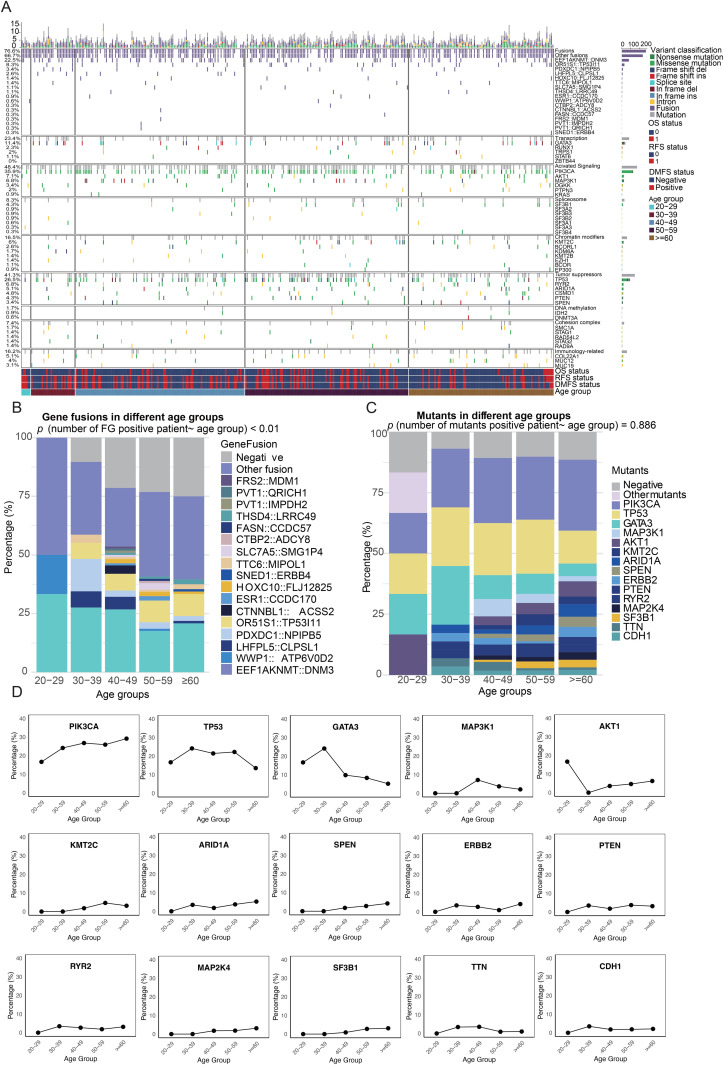
Decreasing FG frequency with advancing age against an age-stable mutational burden in luminal BC in FUSCC-BRCA cohort. (A) Genomic landscape of patients with luminal BC including clinical features, FGs, and genetic mutations. Multicolored bars mapped the different types of gene mutations, representing the number of gene mutations. The percentage on the right indicated the genomic events’ rates of genes in all patients. (B) The bar plot showed the percentage difference of specific FGs among age groups. (C) Differential prevalence of specific gene mutations among age groups. (D) Dot plots showed the frequency of gene mutation in different age groups.

This study identified age-dependent prevalence patterns of two major genomic alterations in luminal BC: FGs and sequence-level mutations. We comprehensively compared the genomic landscape and clinical relevance across different patient age groups (Fig 2A). Tumor mutation burden (TMB), a marker of genomic instability, showed a significant positive correlation with increasing age (S2H Fig). FGs were detected in 76.6% of the overall luminal BC cohort. Notably, patients harboring FGs exhibited poorer prognoses than those without FGs (S3A-C Fig).

Distinct differences in genomic features were observed across age groups. The prevalence of FGs in luminal BC declined significantly with age (*p* < 0.01), with the highest detection rate observed in younger patients (100% among those in their 20s) and the lowest in older individuals (75% among patients in their 60s). A few rare fusion events showed the opposite trend, increasing in prevalence with age (Figs 2B and S4A). Among all detected FGs, the most frequent, *ED* fusion, was most common in young patients (S5 Fig), while most other FGs followed a similar pattern of decreasing frequency with increasing age.

In contrast, the overall frequency of gene mutations did not significantly differ across age groups (Figs 2C, and S4B). The general distribution of somatic mutations remained stable, likely reflecting the dominance of the HR^+^ subtype, which usually shows a consistent mutational profile involving genes such as *CDH1* and *MAP3K1*. However, several mutations demonstrated distinct age-dependent accumulation patterns. Mutations in *PIK3CA*, *ARID1A*, and *SPEN* increased steadily after age 40, whereas *TP53* and *GATA3* mutations decreased markedly after age 30 (Figs 2D and S4C). Interestingly, *PTEN* mutations displayed a biphasic distribution, with incidence peaks during the third and fifth decades of life. In later life stages, elderly patients showed a gradual but statistically nonsignificant increase in the total number of mutations. This modest trend may reflect reduced DNA repair capacity with age, although the overall mutation accumulation rate appeared lower than expected, potentially due to metabolic slowing in older individuals (Fig 2C, D).

### 3.3 Association of FG positivity with elevated mutational burden and its age-dependency in luminal BC

**Fig 3:**
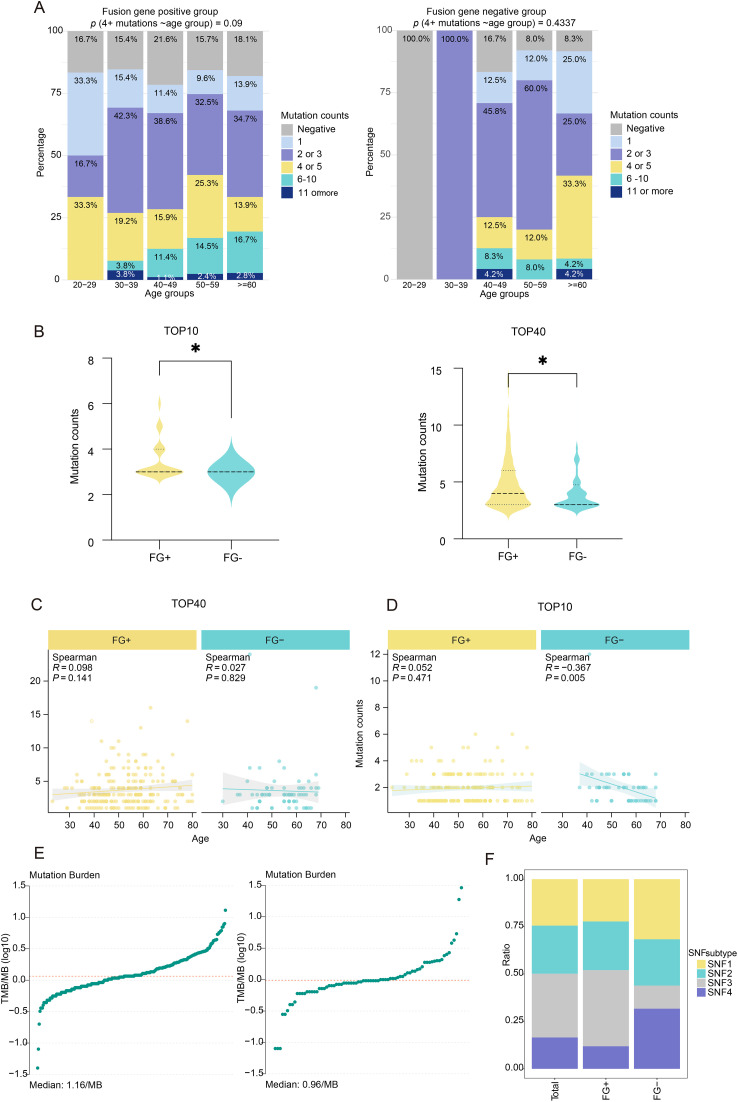
Convergence of FG positivity and mutational burden shapes age-dependent tumorigenesis in luminal BC in FUSCC-BRCA cohort. (A) FG+ patients exhibited a higher prevalence of tumors harboring four or more mutations, as shown by the percentage bar graphs comparing mutation burden categories between FG+ (left) and FG- (right) cohorts. (B) Comparations of mutational burden stratified by FG status. (C) Association between the total number of mutations and age (scatter plot). Spearman correlations were shown on the top. Regression line and the CI were shown. The panels showed top 40 gene mutation items with age-related mutations. (D) Scatter plot of mutation number and age. The panels showed top 10 gene mutation items with age-related mutations. (E) Scatter plots displayed the distribution of tumor mutational burden (TMB) in FG+ (left) and FG- (right) populations. Each point represented one patient, with points arranged from bottom-left to top-right in order of increasing patient age. (F) The percentage bar chart illustrated the distribution of SNF subtypes within the overall population and the FG+ and FG- subgroups.

We next evaluated the relationship between FG status and mutation burden, stratified by age. Among the 40 most recurrent mutations identified in luminal BC, 27% of FG– patients and 33% of FG+ patients across all age groups harbored ≥4 mutations (Figs 3A and S6A-D). FG+ cases consistently exhibited a higher prevalence of elevated mutational burden (defined as ≥3 mutations) compared with FG– cases, both within the top 40 and top 10 most frequent somatic mutations (Figs 3B and S6E, F). A weak but nearly significant age-associated increase in mutation number was observed specifically among FG+ patients (Figs 3C, D and S6H-I). Correlation analysis between TMB and patient age further supported this trend, demonstrating greater mutation accumulation within the FG+ subgroup (Figs 3E, S2H, S6G). When stratified by FUSCC molecular subtype, FG+ luminal tumors exhibited a higher proportion of the SNF3 subtype and a lower proportion of the SNF4 subtype (Fig 3F), suggesting potential subtype-specific enrichment related to FG status.

### 3.4 Transcriptome and proteome correlation analysis across age groups in luminal BC

**Fig 4:**
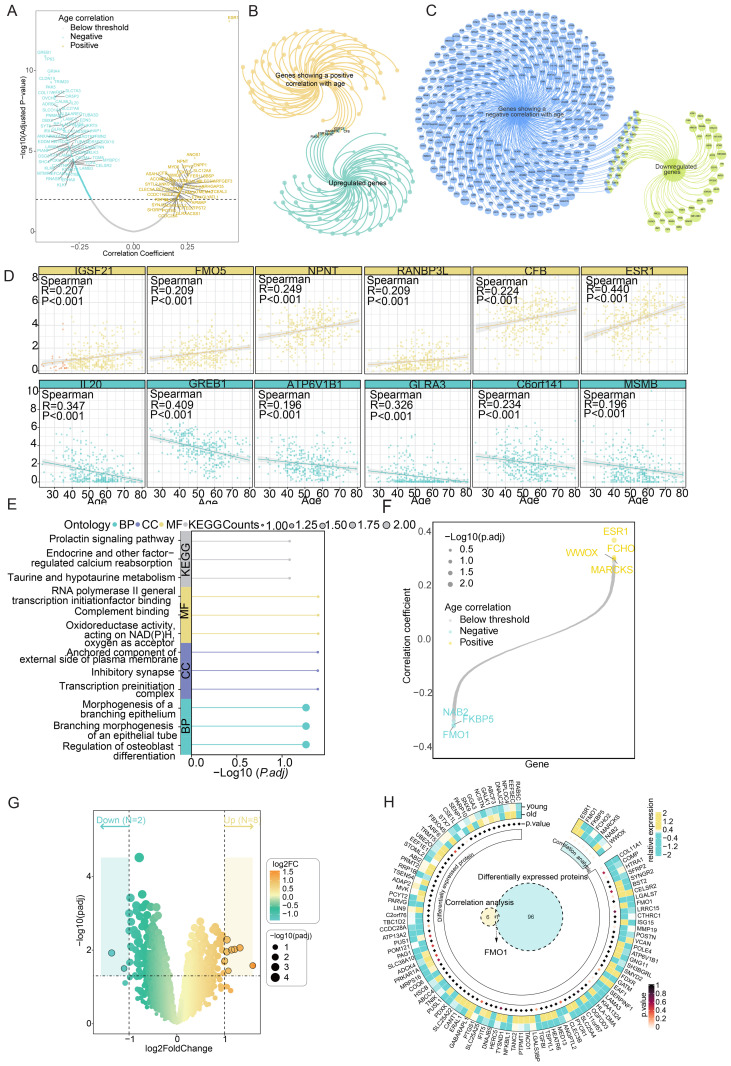
Transcriptomic and proteomic profiling of age-associated genes and pathways in luminal BC. (A) Correlation volcano plots visualized associations between gene expression patterns and patient age. Significantly correlated genes (adjusted *p* < 0.01) are highlighted in orange for positive correlations (Pearson r > 0) and cyan for negative correlations (Pearson r < 0). (B) The panel illustrated the intersection between up-regulated DEGs and age-positively correlated genes. (C) The intersection between down-regulated DEGs and age-negatively correlated genes. (D) Scatter plots of six age-positively and six age-negatively correlated candidate genes from the overlapping set. (E) GO and KEGG enrichment analysis of up-regulated genes from the intersection. (F) The scatter plot of the correlation between protein expression levels and age. (G) The volcano plot of age-related DEPs. (H) The annular heat map and venn diagram jointly presented the intersection and expression distribution of DEPs and age-related proteins.

Genome-wide transcriptomic profiling identified 57 genes positively correlated and 400 genes negatively correlated with advancing age (adjusted *p* < 0.01) (Fig 4A). Consistent age-related expression patterns were validated through merged differentially expressed genes (DEGs) analysis across age cohorts (|logFC| > 0.5 and *p* < 0.05). The overlap between rigorously filtered differentially expressed genes (DEGs) and age-dependent transcriptional changes revealed several biologically relevant candidates. Among them, *FMO5*, *NPNT*, *RANBP3L*, *CFB*, and *ESR1* were confirmed as established biomarkers associated with aging-related luminal BC and poor prognosis (Fig 4B). In addition, *IGSF21* was identified as a novel age-related gene. Integrated transcriptomic and correlation analyses identified additional genes negatively associated with age, including *CELSR2*, *PDZK1*, *C1orf226*, *ADORA1*, *IL20*, *GREB1*, *ATP6V1B*, *TUBA3E*, *TUBA3D*, *GLRA3*, *C6orf141*, *TFPI2*, *SLC7A2*, *PTGES*, *OLFM1*, *PRSS23*, *MSMB*, *RAPGEFL1*, *LAMA3*, *PGLYRP2*, *PHF21B*, *FAM3B*, and *TFF1* (Fig 4C). Further filtering prioritized 12 high-confidence age-related genes, six positively and six negatively correlated with age, after Benjamini–Hochberg adjustment (*p* < 0.0001) (Fig 4D). This combined approach, incorporating DEGs and correlation-based analyses, highlights a biologically robust subset of age-associated candidates for future experimental validation.

Gene set enrichment analysis (GSEA) of overlapping DEGs identified several pathways previously linked to aging and luminal BC biology, including the prolactin signaling pathway, endocrine and calcium reabsorption processes, and RNA polymerase II transcription initiation factor binding (Fig 4E). GSEA of overlapping DEGs identified several pathways previously linked to aging and luminal BC biology, including the prolactin signaling pathway, endocrine and calcium reabsorption processes, and RNA polymerase II transcription initiation factor binding.

To further characterize age-related molecular changes, proteomic profiling was incorporated into the analysis. Several proteins, including ESR1, demonstrated a positive correlation with age, whereas FCHO2, MARCKS, WWOX, NAB2, FKBP5, and FMO1 showed negative correlations (Fig 4F). Protein-level differential expression was assessed using the *limma* package (Fig 4G). Integration of correlation and differential expression analyses identified a single overlapping protein, FMO1, which has not previously been associated with age of onset in BC (Fig 4H).

### 3.5 The interplay between FGs and co-occurring genomic alterations defines divergent clinical outcomes in luminal BC

**Fig 5:**
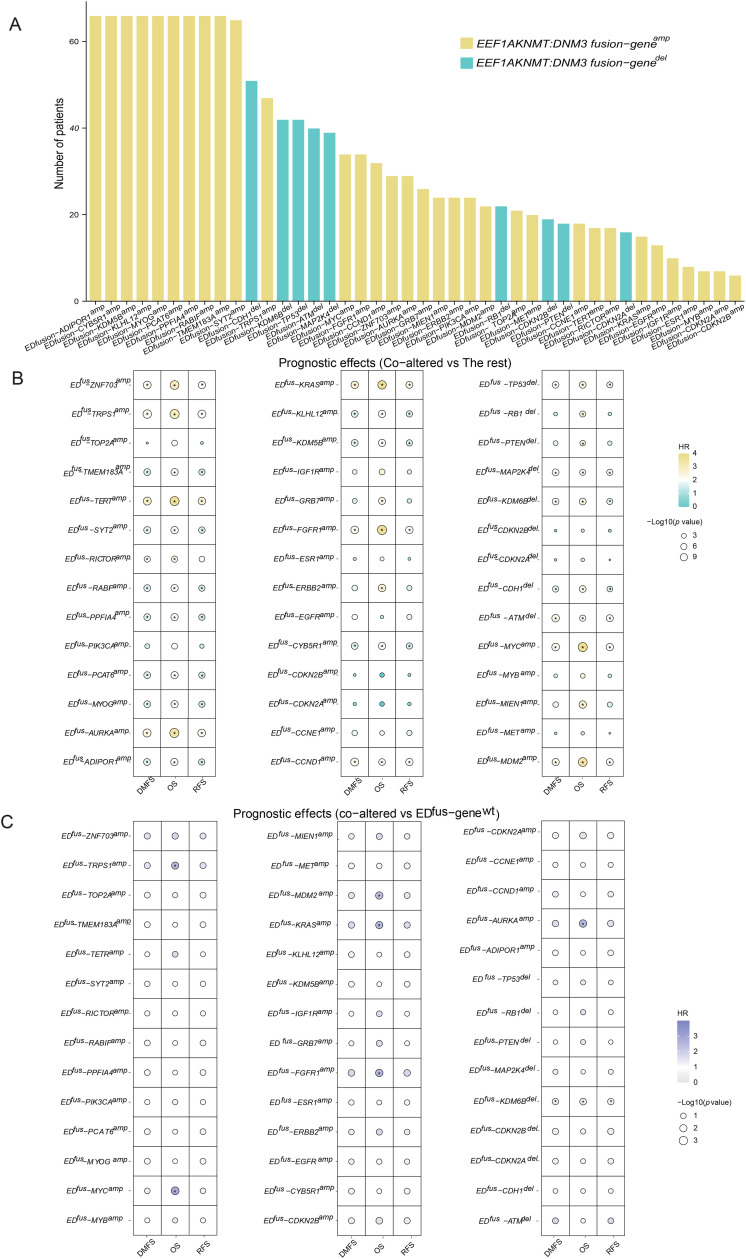
Associations between co-alterations and patient outcomes. (A) Frequency of the top co-alterations of *EEF1AKNMT::DNM3* (*ED*) fusion and gene amplification (*gene*^*amp*^) and gene deletion (*gene*^*del*^). (B) Association between co-alterations and patient survival. Hazard ratios (HRs) were calculated using the Cox proportional hazards model and visualized on a continuous color gradient. Survival differences between groups were assessed using the log-rank test, with significant associations highlighted (*p < 0.05). (C) Association between co-alterations and patient survival between co-occurrence of *ED*^*fus*^*-gene*^*amp/de*l^ versus the *ED*^*fus*^*-gene*^*WT*^. Hazard ratios (HRs) were calculated using the Cox proportional hazards model and visualized on a continuous color gradient. Survival differences between groups were assessed using the log-rank test, with significant associations highlighted (*p < 0.05).

Given that *ED* fusion was the most prevalent fusion event identified, the subsequent step involved examining its interaction with other functional genomic alterations. Tumors exhibiting *ED* fusions frequently demonstrated concomitant copy number amplifications and deletions. Among the leading amplification events associated with the *ED* fusion, *ADIPOR1*, *CYB5R1*, *KDM6B*, *KLHL12*, and five additional genes were the most frequently observed (Fig 5A). Importantly, deletions of *CDH1* and *KDM6B* also occurred with high frequency in cases exhibiting simultaneous copy number loss and *ED* fusion (Fig 5A). To determine the clinical relevance of these co-alterations, we evaluated their association with patient prognosis, focusing on DMFS and OS (Fig 5B). Using a Cox proportional hazards model, we compared outcomes between tumors with and without specific co-alterations. Several co-occurring genomic events, including *ED*^*fus*^*-TRET*^*amp*^, *ED*^*fus*^*-AURKA*^*amp*^, *ED*^*fus*^*-KRAS*^*amp*^, *ED*^*fus*^*-FGFR1*^*amp*^, *ED*^*fus*^*-KDM6B*^*del*^, *ED*^*fus*^*-MYC*^*del*^, and *ED*^*fus*^*-MDM2*^*del*^, were significantly associated with poorer DMFS, OS, and RFS, each with hazard ratios >2 (Fig 5B). To isolate the prognostic contribution of individual co-alterations from the effect of the *ED* fusion itself, we compared each co-alteration to *ED*^*fus*^*-gene*^*WT*^ cases. Remarkably, only *ED*^*fus*^*-KDM6B*^*del*^ remained significantly associated with all three survival endpoints (Fig 5C). Notably, when examining the age distribution of patients with *KDM6B* deficiency, we observed no preferential association with younger age, in contrast to the pattern seen with *ED* fusion events. Instead, the incidence of *KDM6B* deficiency followed a bell-shaped distribution across age groups, with the highest frequency in patients aged 40–60 years and a median age of 51.8 ± 11.3 years, indicating that this co-alteration is not driven by age-related bias. These finding underscored the robustness of the *ED*^*fus*^*-KDM6B*^*del*^ event as an independent prognostic marker in luminal BC. Collectively, these analyses demonstrate that among the various co-alterations assessed, *ED*^*fus*^*-KDM6B*^*del*^ consistently showed the strongest and most independent association with adverse clinical outcomes, including DMFS, RFS, and OS.

### 3.6 *ED*^fus^*-KDM6B*^del^ as a marker of poor prognosis in luminal BC

**Fig 6:**
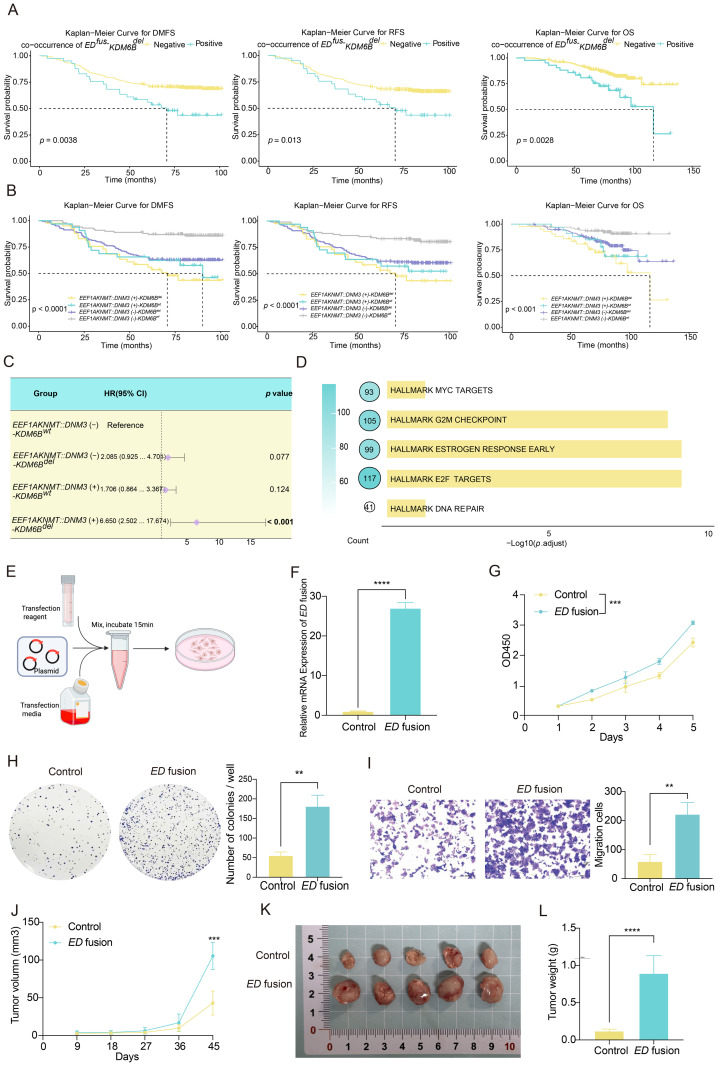
*ED* fusion conferred poor prognosis and promoted proliferation/migration of ER^+^ cancer cells. (A) KM curves of DMFS, RFS, and OS for *ED* fusion-positive patients versus other patients. Survival differences between groups were assessed using the log-rank test. (B) KM curves of DMFS, RFS, and OS for patients with *ED*^*fus*^*-KDM6B*^*del*^ versus patients with or without *ED*^*fus*^*/ KDM6B*^*del*^. Survival differences between groups were assessed using the log-rank test. (C) The *ED* fusion could be regarded as an independent prognostic factor for predicting DMFS in the multivariate regression analysis. The analysis was performed using the Cox proportional hazards model. (D) Enrichment analysis of the *ED*^*fus*^*-KDM6B*^*del*^ positive ones versus the negative ones. GSEA was performed to identify differentially enriched pathways. (E) Transfection of overexpression plasmid (the figure was drawn by the BioRender site). (F) Overexpression efficiency after transfection of MCF-7 cells was validated by qRT-PCR (n = 3). Data are presented as mean ± SEM; statistical significance was determined using the t-test (****p < 0.0001). (G) The CCK-8 assay results of overexpressing the *ED*-FG in MCF-7 cells (n = 3). Data are presented as mean ± SEM; statistical significance was determined using the t-test (***p < 0.001). (H) The colony formation assay of overexpressing the *ED*-FG in MCF-7 cells (n = 3). Statistical significance was determined using the t-test (**p < 0.01). (I) Cell migration was assessed by transwell assay following overexpression of the *ED*-FG in MCF-7 cells (n = 3). Scare bar, 200 μm. Statistical significance was determined using the t-test (**p < 0.01). (J-L) *ED* fusion overexpression promoted tumor growth in vivo. MCF-7 cells were stably transfected with lentiviral vectors carrying either an empty vector control or a plasmid for *ED* fusion overexpression. 1 × 10^6^ MCF-7 cells were injected into each mouse (n = 5 per group). Tumor sizes were measured every nine days until the end of the experiment. Tumor volumes were compared between the *ED* fusion overexpression group and the control group using the t-test (***p < 0.001). At the end of the experiment, tumor weights were measured and compared between the two groups using the t-test (**** *p* < 0.0001).

Building on previous analyses, the co-occurrence of *ED*^*fus*^*-KDM6B*^*del*^ was identified as a marker of poor clinical outcomes in luminal BC. Patients with the *ED*^*fus*^*-KDM6B*^*del*^ alteration showed significantly worse prognoses than non-carriers (Fig 6A). Among all wild-type subgroups, *ED*^*fus*^*-KDM6B*^*del*^ consistently indicated the poorest survival outcomes, including DMFS, RFS, and OS (Fig 6B). As expected, multivariate regression analysis confirmed that *ED*^*fus*^*-KDM6B*^*del*^ functions as an independent prognostic factor for DMFS (Fig 6C). To explore potential mechanisms behind this association, we performed pathway enrichment analysis. The results revealed significant upregulation of proliferative signaling pathways, including MYC targets, G2M checkpoint, E2F targets, and estrogen response pathways, in *ED*^*fus*^*-KDM6B*^*del*^ positive cases (Fig 6D).

Next, we verified the existence of the *ED* fusion using BC samples (S7 Fig). The *ED* fusion was cloned into an overexpression plasmid and transfected into ER^+^ BC cells (Fig 6E). Functional assays demonstrated that *ED* fusion overexpression enhanced cellular proliferation, as evidenced by CCK-8 and colony formation assays (Fig 6F, G). Furthermore, Transwell migration assays confirmed that *ED* fusion overexpression significantly promoted the migratory capacity of ER^+^ cancer cells (Fig 6I). In addition, we further utilized xenograft mice models to investigate the role of *ED* fusion in the regulation of tumor growth in vivo. The results illustrated that *ED* fusion overexpression facilitated the breast tumor growth markedly (Fig 6J-L). Specifically, GSEA was performed to compare pathway enrichment between *ED*^*fus*^*-KDM6B*^*del*^ co-altered tumors and tumors with *ED*^*fus*^ alone. The results showed that co-altered tumors were significantly enriched in pathways associated with cell cycle progression (S8A, B Fig).

## 4 Discussion

Early-onset BC is consistently associated with poorer prognosis [18], particularly within the luminal subtype, even under contemporary treatment regimens [19]. However, the genomic alterations underlying the divergent clinical behavior between young and older patients remain incompletely characterized. In this study, we comprehensively examined multiple molecular factors, including FGs, somatic mutations, and CNVs, to investigate how age influences tumor biology in BC. Through integrated multi-omics analysis, we identified the co-occurrence of *ED*^*fus*^*-KDM6B*^*del*^ as a novel prognostic biomarker associated with aggressive tumor behavior and poor clinical outcomes in the luminal subtype. Moreover, we revealed an age-dependent pattern of genomic remodeling, where younger patients exhibited a higher fusion burden, distinct mutational profiles, and transcriptional reprogramming, which may collectively contribute to high-risk clinical phenotypes.

Somatic mutations in breast epithelial cells accumulate progressively with age, and the number of mutations in mammary tissue increases steadily over time. On average, normal breast epithelial cells acquire approximately 20 new single-nucleotide variants per genome each year. By age 70, the number of coding-region mutations typically ranges from several dozen to several hundred, accounting for 1–2% of total genome-wide mutations, depending on the extent of clonal selection [20,21]. Consistent with this background accumulation, our findings demonstrate that FG frequency is significantly higher in younger patients, with this pattern most robustly observed in the luminal subtype.

Biologically, youth represent a convergence of several active physiological processes, including a hormone-rich microenvironment, elevated cell proliferation rates, and a greater dependence on error-prone DNA repair pathways. Together, these factors may create a permissive context for FG formation, rendering it a dominant oncogenic mechanism in younger patients with luminal BC. In contrast, other BC subtypes may be driven by alternative forms of genomic instability that obscure age-related differences in fusion frequency. Importantly, gene fusions have been implicated in numerous epithelial-derived malignancies, where they are frequently associated with poor prognosis [22–24] and remain challenging targets for precision therapeutics. Consistent with our results, accumulating evidence suggests that younger patients are more prone to FG events [25], particularly those involving genes encoding kinases or transcription factors [26]. These fusions may facilitate tumor progression by activating oncogenic signaling networks and transcriptional deregulation [15,27].

Recent studies increasingly demonstrate a strong association between FG detection and the potential for targeted therapeutic interventions. In our analysis, FG positivity correlated with higher histological grade, elevated Ki-67 index, and increased tumor burden. Notably, FG+ tumors exhibited lower ER expression compared with FG– controls, suggesting a reduced likelihood of response to endocrine therapy. Consistent with this observation, FG+ patients derived limited clinical benefit from endocrine therapy. These findings align with previous reports, which show that genomic instability is often accompanied by reduced ER activity [28–31]. At the same time, they highlight a paradox within luminal BC: certain high-risk tumors retain partial endocrine sensitivity despite molecular features associated with resistance. In FG+ patients, high mutation burden coexists with diminished responsiveness to endocrine therapy. This resistance may arise not only from tumor-intrinsic factors, such as elevated proliferation and Ki-67 expression, but also from alternative mechanisms. These may include activation of ER-independent signaling pathways, intrinsic resistance programs, or increased intratumoral heterogeneity and immunosuppressive remodeling within the tumor microenvironment. Together, these factors likely reduce the efficacy of endocrine therapy. Accordingly, therapeutic strategies for FG+ patients should extend beyond ER blockade and consider intensified or combination approaches that target both genomic instability and proliferative signaling.

Our data further demonstrate that the co-occurrence of the *ED* fusion and *KDM6B* deletion (*ED*^*fus*^*-KDM6B*^*del*^) consistently predicts poor prognosis across all survival metrics, even after adjustment for clinical covariates. Mechanistically, enrichment analyses suggest that activation of pro-proliferative pathways, including MYC, E2F, and G2M checkpoint targets, drives this aggressive phenotype. We further hypothesize that KDM6B deficiency in ED fusion-positive tumors may relieve the epigenetic repression of cell cycle-related genes, thereby promoting cell cycle progression. This hypothesis is supported by our GSEA findings demonstrating that *ED*^*fus*^*-KDM6B*^*del*^ co-altered tumors are significantly enriched in cell cycle-related pathways compared with *ED*^*fus*^-only tumors. Thus, the concurrent loss of *KDM6B* may amplify the proliferative signals driven by the *ED* fusion, contributing to the aggressive phenotype observed in this subgroup. Future chromatin immunoprecipitation sequencing for H3K27me3 and functional rescue experiments will be essential to validate the direct epigenetic targets of KDM6B in this context. Functional validation confirmed that *ED* fusion overexpression enhances the proliferative capacity of ER^+^ BC cells *in vitro*, supporting its role as an oncogenic driver. To our knowledge, this molecular synergy between *ED* fusion and *KDM6B* loss has not been previously reported in BC. Although *KDM6B* has been characterized as a tumor suppressor in several cancers [32–34], its involvement in fusion-driven transcriptional programs introduces a new dimension to the epigenetic regulation of luminal tumors. Our findings diverge from reports suggesting that *KDM6B* deficiency alone does not strongly influence tumor progression [35]. This apparent discrepancy may reflect the context-dependent nature of *KDM6B* function, as its impact appears to be shaped by concurrent genomic alterations, particularly gene fusions that remodel chromatin accessibility and transcriptional output. From a pathway perspective, *ED*^fus^*-KDM6B*^del^ was significantly enriched in cell cycle–related processes, supporting the hypothesis that it promotes tumor progression through coordinated dysregulation of epigenetic and proliferative signaling. This mechanistic model aligns with the central role of PARP-1 in the DNA damage response and highlights *KDM6B* as a potential predictive biomarker for chemotherapy responsiveness [36].

Previous studies have shown that protein–RNA correlation holds promise as a prognostic biomarker for predicting late recurrence in luminal BC, underscoring the clinical potential of multi-omics integration for risk stratification [4,37,38]. In our analysis, several genes and proteins, including *ESR1* and *FMO1*, were found to be significantly associated with age at both the transcriptomic and proteomic levels. Notably, *FMO1* exhibited an inverse correlation with age, and its role in the aging process has not yet been described in BC, making it a promising candidate for further functional investigation. Prior research has also reported age-dependent differences in the tumor immune landscape, supported by both immunohistochemical and metabolic profiling [39,40]. Specifically, younger patients tend to display a higher density of PD-L1– and OX40-positive tumor-infiltrating lymphocytes, including both T and B cell subsets, compared with older individuals [39]. These findings underscore the importance of employing multi-omics approaches to identify previously unrecognized aspects of tumor biology and immune regulation across various age groups.

Beyond the analysis of age-associated FG prevalence and prognostic impact, our study revealed several novel insights into genomic alterations in luminal BC, some of which diverge from previously published findings. We observed a dual age-related effect on genomic stability: FG prevalence declined with increasing age, whereas TMB showed a modest age-associated rise. This trend was particularly evident in FG+ patients, who exhibited greater overall accumulation of mutations. Such patterns may reflect an age-related decline in DNA repair capacity or evolving tumor microenvironmental conditions, contrasting with earlier studies [41,42]. These discrepancies likely stem from differences in cohort composition or analytical methodologies used to assess genomic instability. Furthermore, we identified distinct age-specific mutational trends, such as *PIK3CA* and *ARID1A* mutations, that accumulated progressively in middle-aged and older patients, whereas *TP53* mutations were more frequent in younger individuals. These findings suggest that different molecular mechanisms predominate across age strata, influencing both tumor behavior and clinical outcomes.

### 4.1 Limitations of the study

The findings of this study should be interpreted in light of certain limitations. The retrospective design may have introduced selection bias, and FG detection by RNA sequencing can be limited in samples with low tumor purity. Additionally, the biological mechanisms underlying the interaction between FGs and co-occurring mutations require further experimental validation to establish causality.

In conclusion, this multi-omics analysis elucidated the age-dependent clinical and molecular relevance of FGs in luminal BC, emphasizing their potential utility in risk stratification and treatment optimization, particularly for younger patients. This study makes two key contributions. First, it identifies age-associated molecular markers, including FGs, that enhance our understanding of how age shapes tumor biology and prognosis, offering new avenues for early prediction of high-risk disease. Second, it broadens the current framework of age-related genomic alterations in BC, advancing efforts toward precision oncology. Future research should focus on developing targeted therapeutic strategies for tumors harboring *ED*^*fus*^*-KDM6B*^*del*^ co-alterations and exploring the potential of FGs as non-invasive biomarkers for early detection and disease monitoring. Overall, our findings provide a novel perspective on the biological and clinical underpinnings of poor prognosis in young patients with BC, highlighting the need for age-informed approaches to precision therapy.

## Supporting information

S1 Fig Distribution and prognostic stratification of age groups in distinct subtype and prognostic significance in the external validation cohort of TCGA-BRCA. Histograms showed population distribution of the overall, FG+ and FG- subgroup respectively in (A) luminal subtype, (B)HER2-enriched subtype, and (C) TNBC subtype. (D) Comparation of the number of FGs between FG+ and FG- patients with BC. (E, F) RFS and OS analysis of luminal BC patients based on age groups.
(PDF)

S2 Fig FG+ luminal tumors derived inferior ET benefit. (A, B) RFS and OS analysis (KM curve) of all luminal BC patients, grouped by whether they received ET. (C, D) RFS and OS analysis of a luminal patients over 40y, grouped by whether they received ET. (E-G) DMFS, RFS and OS analysis of a luminal patients less than 40y, grouped by whether they received ET. (H) Scatter plots displayed the distribution of TMB in total populations.
(PDF)

S3 Fig Survival results between FG+ and FG- group in FUSCC-BRCA cohort. (A-C) DMFS, RFS, and OS analysis of FG+ and FG- patients.
(PDF)

S4 Fig Decreasing FG frequency with advancing age against an age-stable mutational burden in luminal BC in TCGA-BRCA cohort. (A) The bar plot showed the percentage difference of specific FGs among age groups. (B) Differential prevalence of specific gene mutations among age groups. (C) Dot plots showed the sex difference of genetic mutations in different age groups.
(PDF)

S5 Fig Dot plots showed the frequencies of FGs in different age groups. (PDF)

S6 Fig External validation of convergence of FG positivity and mutational burden in TCGA-BRCA cohort. (A, B) In the top 40 mutations’ group, FG+ patients exhibited a higher prevalence of tumors harboring four or more mutations. (C, D) In the top 10 mutations’ group, FG+ patients exhibited a higher prevalence of tumors harboring four or more mutations. (E, G) Comparations of mutational burden stratified by FG status. (H, I) Scatter plot of mutation number and age.
(PDF)

S7 Fig DNA agarose gel imaging of the PCR products of the ED fusion from three different patients of luminal subtype. (PDF)

S8 Fig (A, B) GSEA revealed that *ED^fus^-KDM6B^del^* co-altered tumors were significantly enriched in cell cycle-related pathways, including E2F targets (A) and G2M checkpoint (B), compared with *ED^fus^*-only tumors. NES, normalized enrichment score; FDR, false discovery rate. (PDF)

## References

[1] Bray F, Laversanne M, Sung H, Ferlay J, Siegel RL, Soerjomataram I. Global cancer statistics 2022: GLOBOCAN estimates of incidence and mortality worldwide for 36 cancers in 185 countries. CA Cancer J Clin. 2024;74(3):229–63. doi: 10.3322/caac.21834 38572751

[2] Siegel RL, Giaquinto AN, Jemal A. Cancer statistics, 2024. CA Cancer J Clin. 2024;74(1):12–49. doi: 10.3322/caac.21820 38230766

[3] Loibl S, Poortmans P, Morrow M, Denkert C, Curigliano G. Breast cancer. Lancet. 2021;397(10286):1750–69. doi: 10.1016/S0140-6736(20)32381-3 33812473

[4] Luen SJ, Viale G, Nik-Zainal S, Savas P, Kammler R, Dell’Orto P, et al. Genomic characterisation of hormone receptor-positive breast cancer arising in very young women. Ann Oncol. 2023;34(4):397–409. doi: 10.1016/j.annonc.2023.01.009 36709040 PMC10619213

[5] Walbaum B, García-Fructuoso I, Martínez-Sáez O, Schettini F, Sánchez C, Acevedo F, et al. Hormone receptor-positive early breast cancer in young women: A comprehensive review. Cancer Treat Rev. 2024;129:102804. doi: 10.1016/j.ctrv.2024.102804 39084152

[6] Saha P, Regan MM, Pagani O, Francis PA, Walley BA, Ribi K, et al. Treatment Efficacy, Adherence, and Quality of Life Among Women Younger Than 35 Years in the International Breast Cancer Study Group TEXT and SOFT Adjuvant Endocrine Therapy Trials. J Clin Oncol. 2017;35(27):3113–22. doi: 10.1200/JCO.2016.72.0946 28654365 PMC5597253

[7] Azim HA Jr, Nguyen B, Brohée S, Zoppoli G, Sotiriou C. Genomic aberrations in young and elderly breast cancer patients. BMC Med. 2015;13:266. doi: 10.1186/s12916-015-0504-3 26467651 PMC4606505

[8] Waks AG, Kim D, Jain E, Snow C, Kirkner GJ, Rosenberg SM, et al. Somatic and Germline Genomic Alterations in Very Young Women with Breast Cancer. Clin Cancer Res. 2022;28(11):2339–48. doi: 10.1158/1078-0432.CCR-21-2572 35101884 PMC9359721

[9] Mitelman F, Johansson B, Mertens F. The impact of translocations and gene fusions on cancer causation. Nat Rev Cancer. 2007;7(4):233–45. doi: 10.1038/nrc2091 17361217

[10] Jin X, Zhou Y-F, Ma D, Zhao S, Lin C-J, Xiao Y, et al. Molecular classification of hormone receptor-positive HER2-negative breast cancer. Nat Genet. 2023;55(10):1696–708. doi: 10.1038/s41588-023-01507-7 37770634

[11] Jiang Y-Z, Ma D, Jin X, Xiao Y, Yu Y, Shi J, et al. Integrated multiomic profiling of breast cancer in the Chinese population reveals patient stratification and therapeutic vulnerabilities. Nat Cancer. 2024;5(4):673–90. doi: 10.1038/s43018-024-00725-0 38347143

[12] Lin C-J, Jin X, Ma D, Chen C, Ou-Yang Y, Pei Y-C, et al. Genetic interactions reveal distinct biological and therapeutic implications in breast cancer. Cancer Cell. 2024;42(4):701-719.e12. doi: 10.1016/j.ccell.2024.03.006 38593782

[13] Liu C, Sun L, Niu N, Hou P, Chen G, Wang H, et al. Molecular classification of hormone receptor-positive /HER2-positive breast cancer reveals potential neoadjuvant therapeutic strategies. Signal Transduct Target Ther. 2025;10(1):97. doi: 10.1038/s41392-025-02181-3 40133264 PMC11937365

[14] Bassez A, Vos H, Van Dyck L, Floris G, Arijs I, Desmedt C, et al. A single-cell map of intratumoral changes during anti-PD1 treatment of patients with breast cancer. Nat Med. 2021;27(5):820–32. doi: 10.1038/s41591-021-01323-8 33958794

[15] Li J-F, Cheng W-Y, Lin X-J, Wen L-J, Wang K, Zhu Y-M, et al. Aging and comprehensive molecular profiling in acute myeloid leukemia. Proc Natl Acad Sci U S A. 2024;121(10):e2319366121. doi: 10.1073/pnas.2319366121 38422020 PMC10927507

[16] Hermida-Prado F, Jeselsohn R. The ESR1 Mutations: From Bedside to Bench to Bedside. Cancer Res. 2021;81(3):537–8. doi: 10.1158/0008-5472.CAN-20-4037 33526469

[17] Nagaraj G, Ma CX. Clinical Challenges in the Management of Hormone Receptor-Positive, Human Epidermal Growth Factor Receptor 2-Negative Metastatic Breast Cancer: A Literature Review. Adv Ther. 2021;38(1):109–36. doi: 10.1007/s12325-020-01552-2 33190190 PMC7854469

[18] Qu Z, Li Z, Pei S, Lu Y, Liu Q, Ding P, et al. Global, Regional, and National Burden of Breast Cancer in Adolescents and Young Adults Aged 15-39 Years From 1990 to 2021 Based on the Global Burden of Disease Study 2021. Cancer Innov. 2025;4(4):e70016. doi: 10.1002/cai2.70016 40487561 PMC12142427

[19] Shin DS, Lee J, Kang E, Noh D, Cheun J-H, Lee J-H, et al. Age and Late Recurrence in Young Patients With ER-Positive, ERBB2-Negative Breast Cancer. JAMA Netw Open. 2024;7(11):e2442663. doi: 10.1001/jamanetworkopen.2024.42663 39509133 PMC11544499

[20] Alexandrov LB, Jones PH, Wedge DC, Sale JE, Campbell PJ, Nik-Zainal S, et al. Clock-like mutational processes in human somatic cells. Nat Genet. 2015;47(12):1402–7. doi: 10.1038/ng.3441 26551669 PMC4783858

[21] Sun S, Brazhnik K, Lee M, Maslov AY, Zhang Y, Huang Z, et al. Single-cell analysis of somatic mutation burden in mammary epithelial cells of pathogenic BRCA1/2 mutation carriers. J Clin Invest. 2022;132(5):e148113. doi: 10.1172/JCI148113 35025760 PMC8884908

[22] Drilon A, Laetsch TW, Kummar S, DuBois SG, Lassen UN, Demetri GD, et al. Efficacy of Larotrectinib in TRK Fusion-Positive Cancers in Adults and Children. N Engl J Med. 2018;378(8):731–9. doi: 10.1056/NEJMoa1714448 29466156 PMC5857389

[23] Gautschi O, Milia J, Filleron T, Wolf J, Carbone DP, Owen D, et al. Targeting RET in Patients With RET-Rearranged Lung Cancers: Results From the Global, Multicenter RET Registry. J Clin Oncol. 2017;35(13):1403–10. doi: 10.1200/JCO.2016.70.9352 28447912 PMC5559893

[24] Li AY, McCusker MG, Russo A, Scilla KA, Gittens A, Arensmeyer K, et al. RET fusions in solid tumors. Cancer Treat Rev. 2019;81:101911. doi: 10.1016/j.ctrv.2019.101911 31715421

[25] Demicco EG, Wagner MJ, Maki RG, Gupta V, Iofin I, Lazar AJ, et al. Risk assessment in solitary fibrous tumors: validation and refinement of a risk stratification model. Mod Pathol. 2017;30(10):1433–42. doi: 10.1038/modpathol.2017.54 28731041

[26] de la Fouchardière A, Pissaloux D, Houlier A, Paindavoine S, Tirode F, LeBoit PE, et al. Histologic and Genetic Features of 51 Melanocytic Neoplasms With Protein Kinase C Fusion Genes. Mod Pathol. 2023;36(11):100286. doi: 10.1016/j.modpat.2023.100286 37474004

[27] Degasperi A, Amarante TD, Czarnecki J, Shooter S, Zou X, Glodzik D, et al. A practical framework and online tool for mutational signature analyses show inter-tissue variation and driver dependencies. Nat Cancer. 2020;1(2):249–63. doi: 10.1038/s43018-020-0027-5 32118208 PMC7048622

[28] Denkert C, Rachakonda S, Karn T, Weber K, Martin M, Marmé F, et al. Dynamics of molecular heterogeneity in high-risk luminal breast cancer-From intrinsic to adaptive subtyping. Cancer Cell. 2025;43(2):232-247.e4. doi: 10.1016/j.ccell.2025.01.002 39933898

[29] Whitworth P, Beitsch P, Mislowsky A, Pellicane JV, Nash C, Murray M, et al. Chemosensitivity and Endocrine Sensitivity in Clinical Luminal Breast Cancer Patients in the Prospective Neoadjuvant Breast Registry Symphony Trial (NBRST) Predicted by Molecular Subtyping. Ann Surg Oncol. 2017;24(3):669–75. doi: 10.1245/s10434-016-5600-x 27770345 PMC5306085

[30] Goetz MP, Toi M, Campone M, Sohn J, Paluch-Shimon S, Huober J, et al. MONARCH 3: Abemaciclib As Initial Therapy for Advanced Breast Cancer. J Clin Oncol. 2017;35(32):3638–46. doi: 10.1200/JCO.2017.75.6155 28968163

[31] Johnston SRD, Toi M, O’Shaughnessy J, Rastogi P, Campone M, Neven P, et al. Abemaciclib plus endocrine therapy for hormone receptor-positive, HER2-negative, node-positive, high-risk early breast cancer (monarchE): results from a preplanned interim analysis of a randomised, open-label, phase 3 trial. Lancet Oncol. 2023;24(1):77–90. doi: 10.1016/S1470-2045(22)00694-5 36493792 PMC11200328

[32] Xun J, Gao R, Wang B, Li Y, Ma Y, Guan J, et al. Histone demethylase KDM6B inhibits breast cancer metastasis by regulating Wnt/β-catenin signaling. FEBS Open Bio. 2021;11(8):2273–81. doi: 10.1002/2211-5463.13236 34165914 PMC8329947

[33] Zhang M, Tang C, Li S, Jiang X, Li B, Chen Y, et al. NSUN2-mediated m5C modification of KDM6B mRNA enhances osteoclast differentiation and promotes breast cancer bone metastasis. Cancer Lett. 2025;631:217939. doi: 10.1016/j.canlet.2025.217939 40692065

[34] Xun J, Du L, Gao R, Shen L, Wang D, Kang L, et al. Cancer-derived exosomal miR-138-5p modulates polarization of tumor-associated macrophages through inhibition of KDM6B. Theranostics. 2021;11(14):6847–59. doi: 10.7150/thno.51864 34093857 PMC8171095

[35] Yang S-G, Li C-P, Wang X-W, Huang T, Qian C, Li Q, et al. Roles of Kdm6a and Kdm6b in Regulation of Mammalian Neural Regeneration. Adv Sci (Weinh). 2025;12(16):e2405537. doi: 10.1002/advs.202405537 39951327 PMC12021076

[36] Yang M, Wang C, Zhou M, Bao L, Wang Y, Kumar A, et al. KDM6B promotes PARthanatos via suppression of O6-methylguanine DNA methyltransferase repair and sustained checkpoint response. Nucleic Acids Res. 2022;50(11):6313–31. doi: 10.1093/nar/gkac471 35648484 PMC9226499

[37] Yoon K-A, Kim Y, Jung S-Y, Ryu J-S, Kim K-H, Lee E-G, et al. Proteogenomic analysis dissects early-onset breast cancer patients with prognostic relevance. Exp Mol Med. 2024;56(11):2382–94. doi: 10.1038/s12276-024-01332-w 39482530 PMC11612404

[38] Angarola BL, Sharma S, Katiyar N, Kang HG, Nehar-Belaid D, Park S, et al. Comprehensive single-cell aging atlas of healthy mammary tissues reveals shared epigenomic and transcriptomic signatures of aging and cancer. Nat Aging. 2025;5(1):122–43. doi: 10.1038/s43587-024-00751-8 39587369 PMC11754115

[39] Hatse S, Lambrechts Y, Antoranz Martinez A, De Schepper M, Geukens T, Vos H, et al. Dissecting the immune infiltrate of primary luminal B-like breast carcinomas in relation to age. J Pathol. 2024;264(3):344–56. doi: 10.1002/path.6354 39344093

[40] Liu W, Wang Y, Bozi LHM, Fischer PD, Jedrychowski MP, Xiao H, et al. Lactate regulates cell cycle by remodelling the anaphase promoting complex. Nature. 2023;616(7958):790–7. doi: 10.1038/s41586-023-05939-3 36921622 PMC12175651

[41] Bai H, Liu X, Lin M, Meng Y, Tang R, Guo Y, et al. Progressive senescence programs induce intrinsic vulnerability to aging-related female breast cancer. Nat Commun. 2024;15(1):5154. doi: 10.1038/s41467-024-49106-2 38886378 PMC11183265

[42] Selenica P, Dopeso H, Repetto M, Basili T, Gazzo AM, Schwartz CJ, et al. Genomic landscape of breast cancer in elderly patients. NPJ Breast Cancer. 2025;11(1):70. doi: 10.1038/s41523-025-00781-4 40640183 PMC12246064

