# Supplementary figures and images for: A fusion-deletion genomic-event underlies poor prognosis in young patients with luminal breast cancer
Source: PLoS One. 2026 Jun 16;21(6):e0349410. doi: 10.1371/journal.pone.0349410 (PMC13271521; doi:10.1371/journal.pone.0349410)

Figure S1

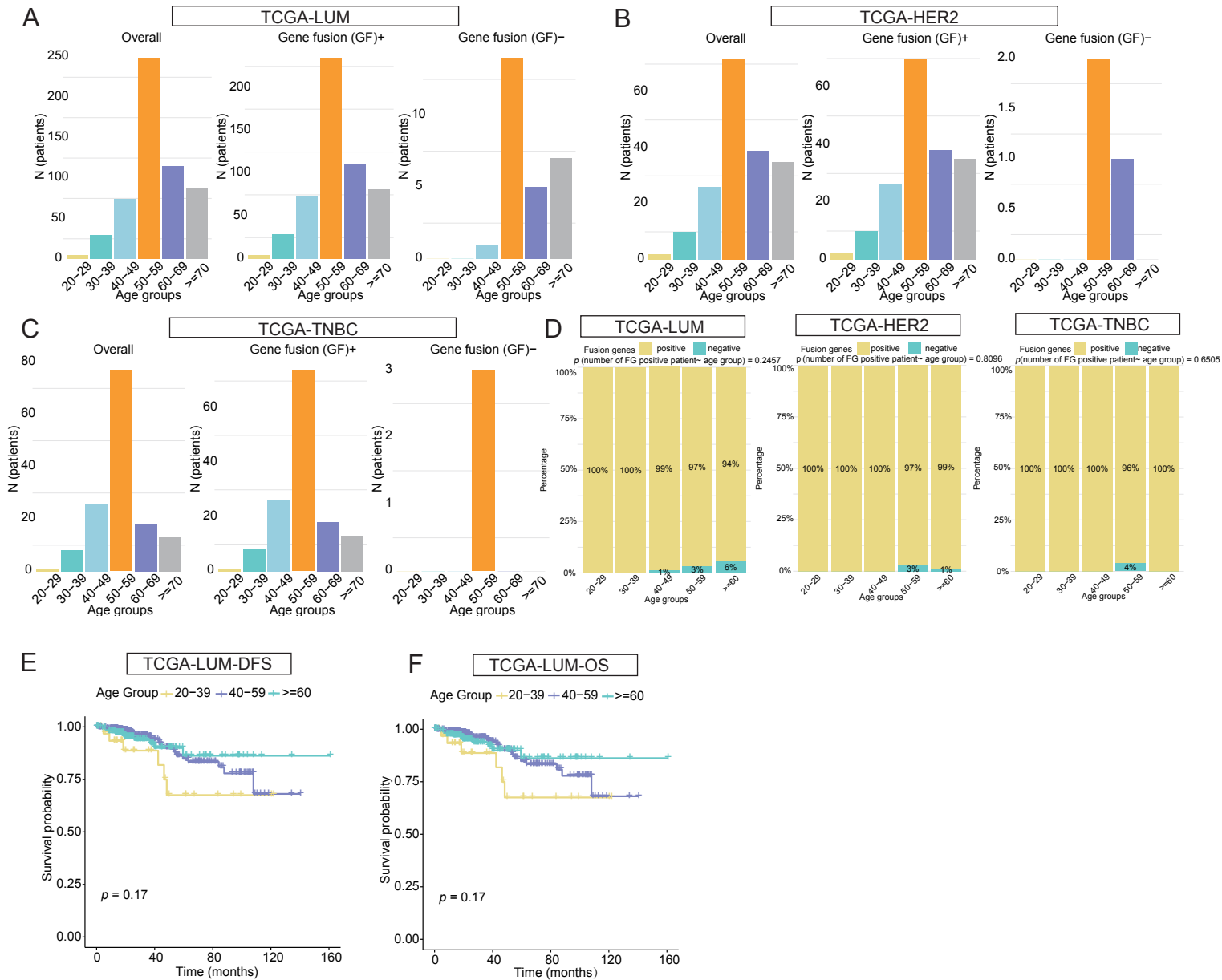

Supplement: S1 Fig — Histograms showed population distribution of the overall, FG+ and FG- subgroup respectively in (A) luminal subtype, (B)HER2-enriched subtype, and (C) TNBC subtype. (D) Comparation of the number of FGs between FG+ and FG- patients with BC. (E, F) RFS and OS analysis of luminal BC patients based on age groups. (PDF) [file pone.0349410.s001.pdf]

Figure S2

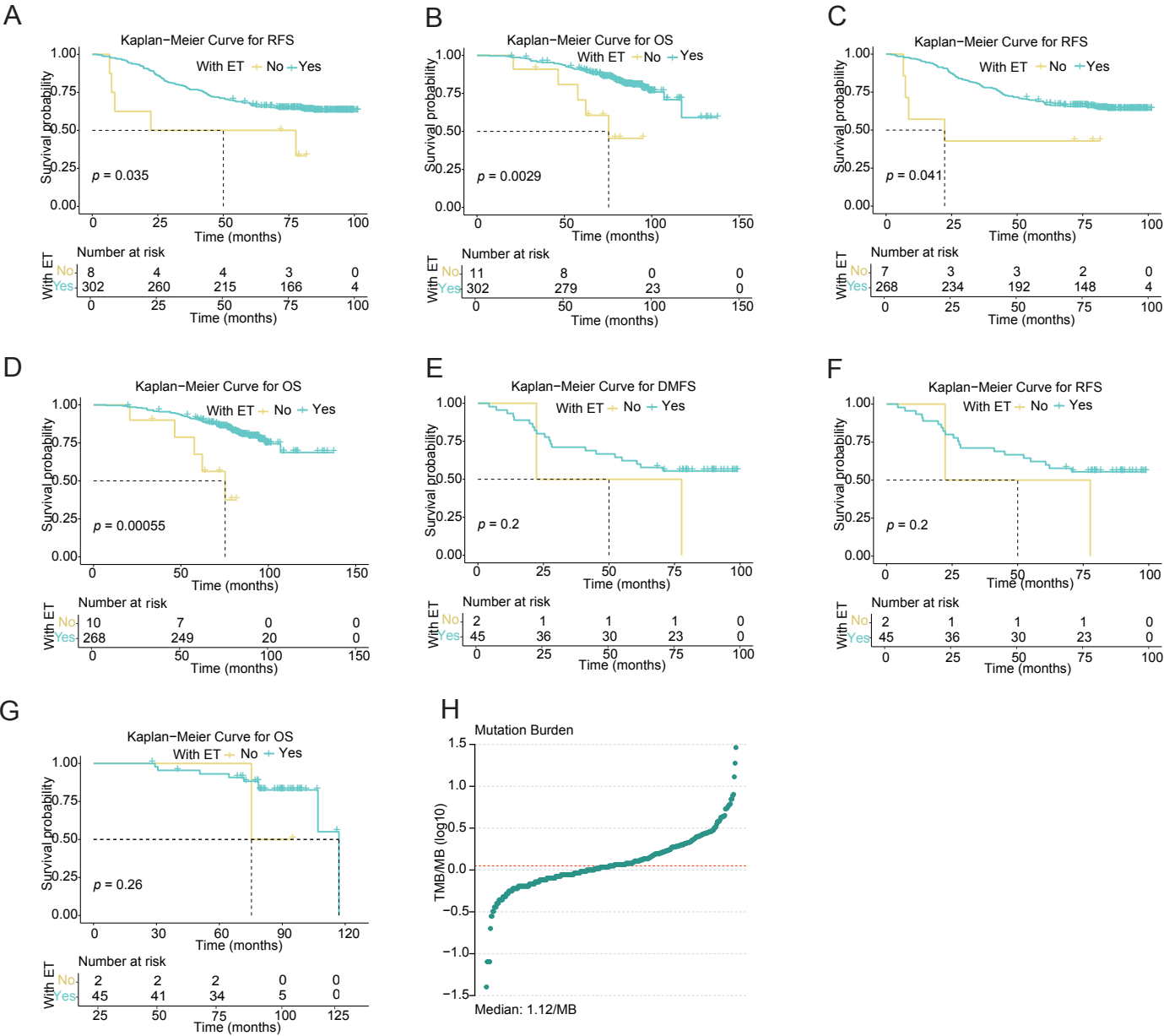

Supplement: S2 Fig — (A, B) RFS and OS analysis (KM curve) of all luminal BC patients, grouped by whether they received ET. (C, D) RFS and OS analysis of a luminal patients over 40y, grouped by whether they received ET. (E-G) DMFS, RFS and OS analysis of a luminal patients less than 40y, grouped by whether they received ET. (H) Scatter plots displayed the distribution of TMB in total populations. (PDF) [file pone.0349410.s002.pdf]

Figure S3

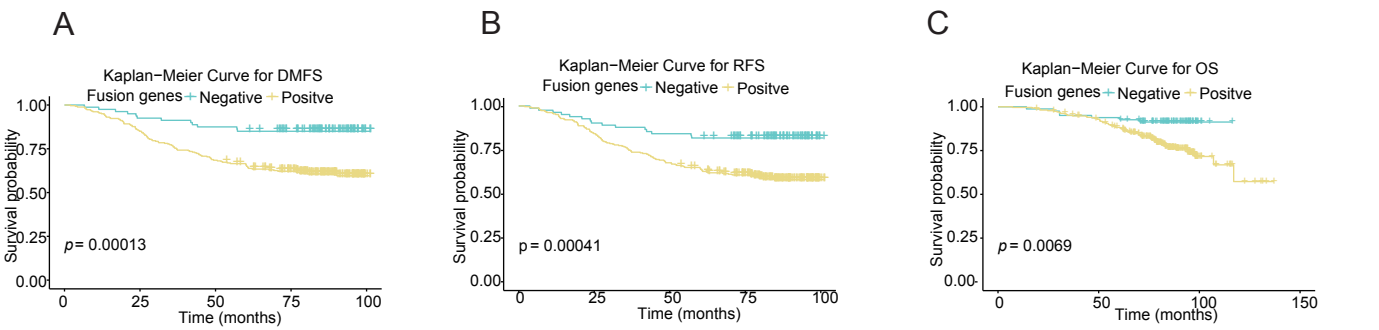

Supplement: S3 Fig — (A-C) DMFS, RFS, and OS analysis of FG+ and FG- patients. (PDF) [file pone.0349410.s003.pdf]

Figure S4

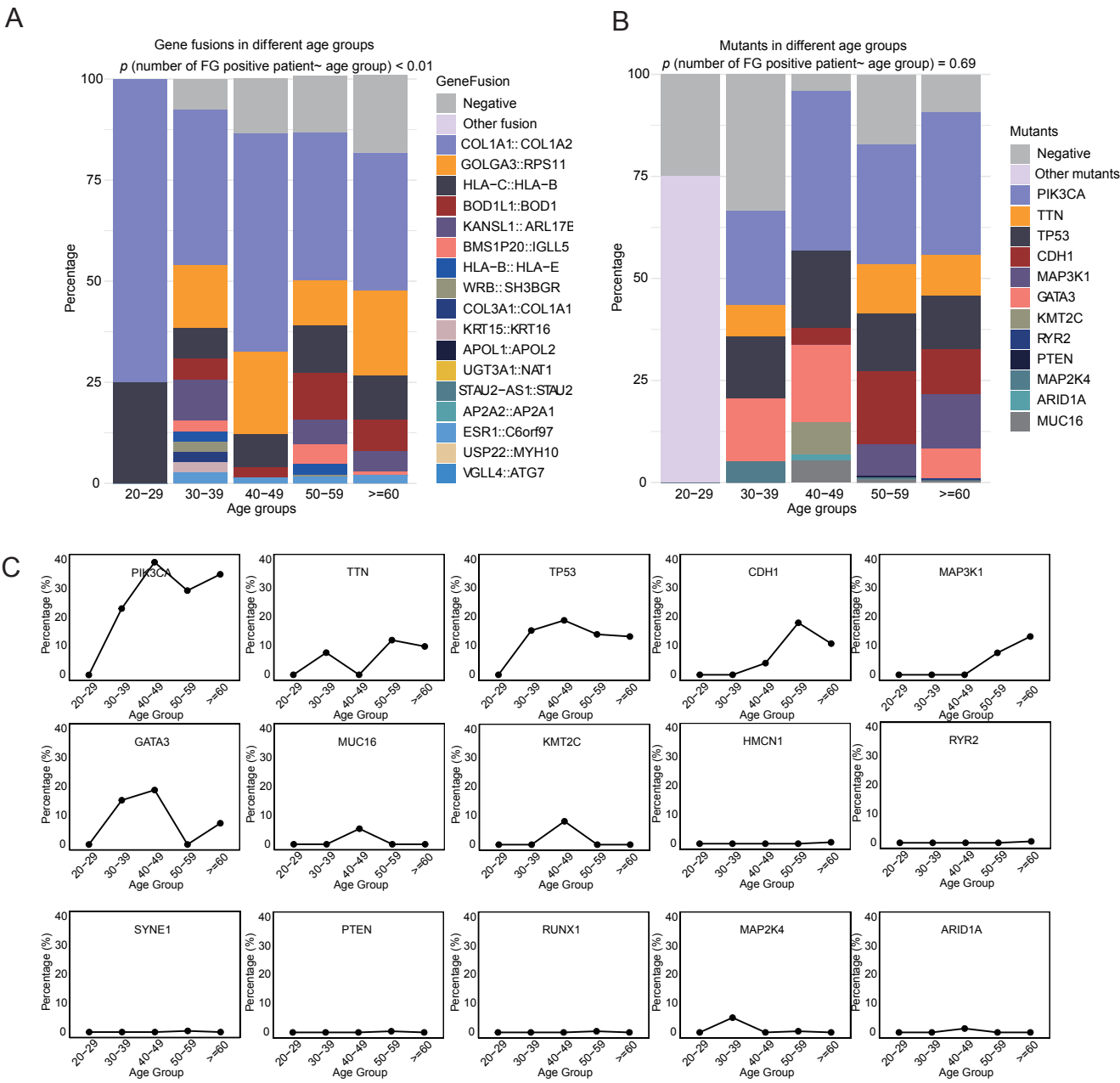

Supplement: S4 Fig — (A) The bar plot showed the percentage difference of specific FGs among age groups. (B) Differential prevalence of specific gene mutations among age groups. (C) Dot plots showed the sex difference of genetic mutations in different age groups. (PDF) [file pone.0349410.s004.pdf]

Figure S5

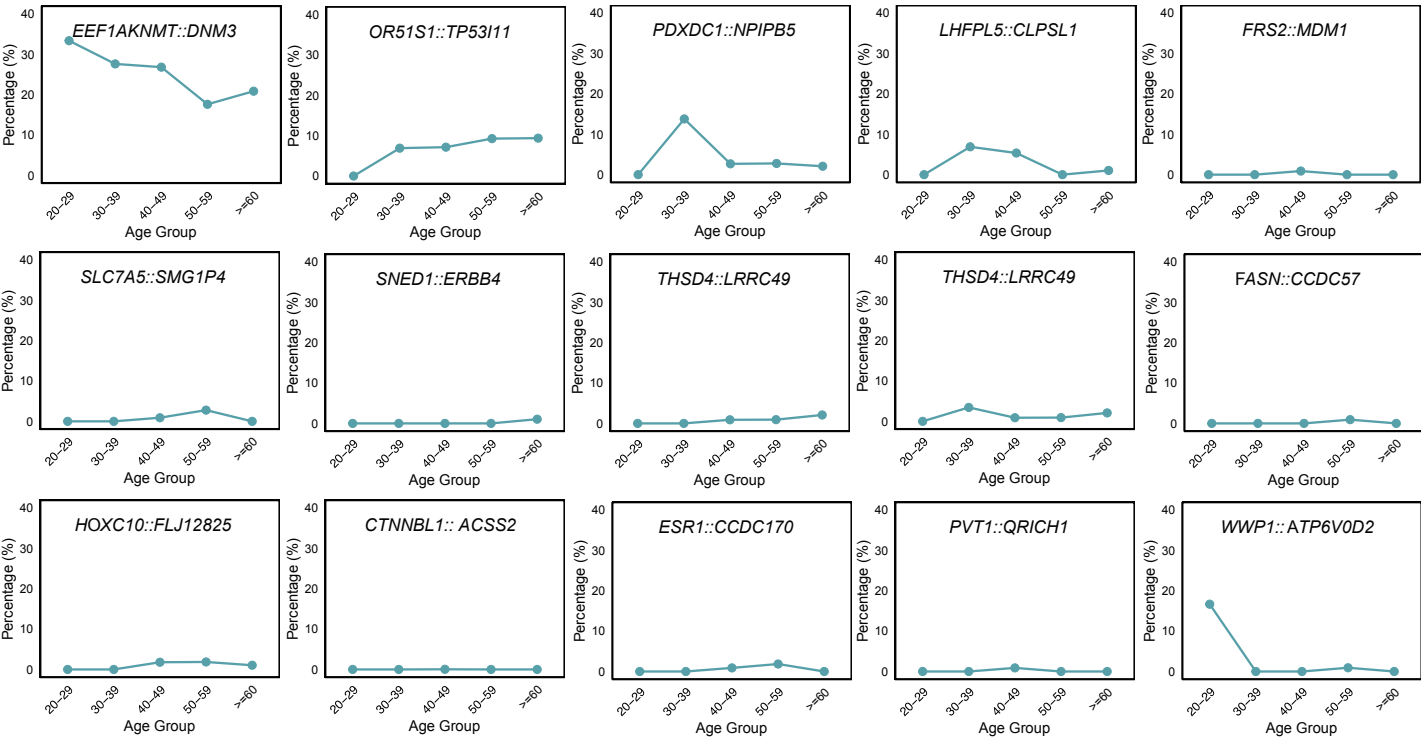

Supplement: S5 Fig — (PDF) [file pone.0349410.s005.pdf]

**Figure S6**

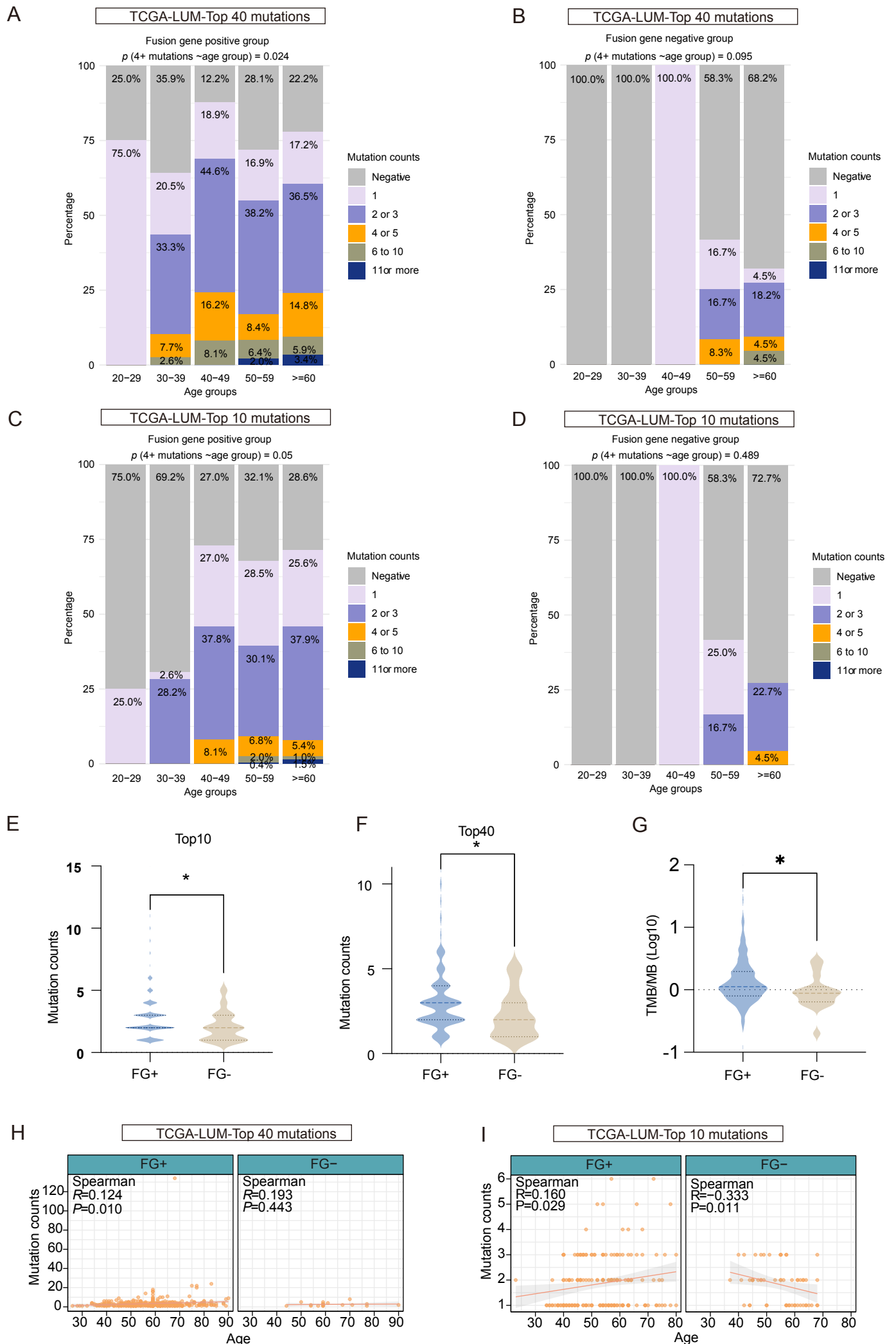

Supplement: S6 Fig — (A, B) In the top 40 mutations’ group, FG+ patients exhibited a higher prevalence of tumors harboring four or more mutations. (C, D) In the top 10 mutations’ group, FG+ patients exhibited a higher prevalence of tumors harboring four or more mutations. (E, G) Comparations of mutational burden stratified by FG status. (H, I) Scatter plot of mutation number and age. (PDF) [file pone.0349410.s006.pdf]

**Figure S7**

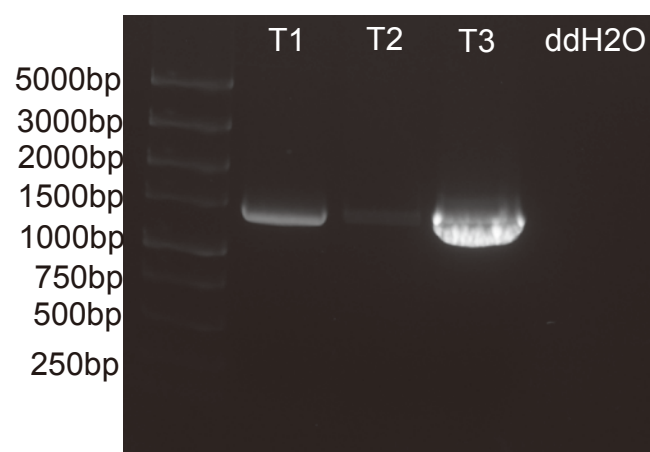

Supplement: S7 Fig — (PDF) [file pone.0349410.s007.pdf]

Figure S8

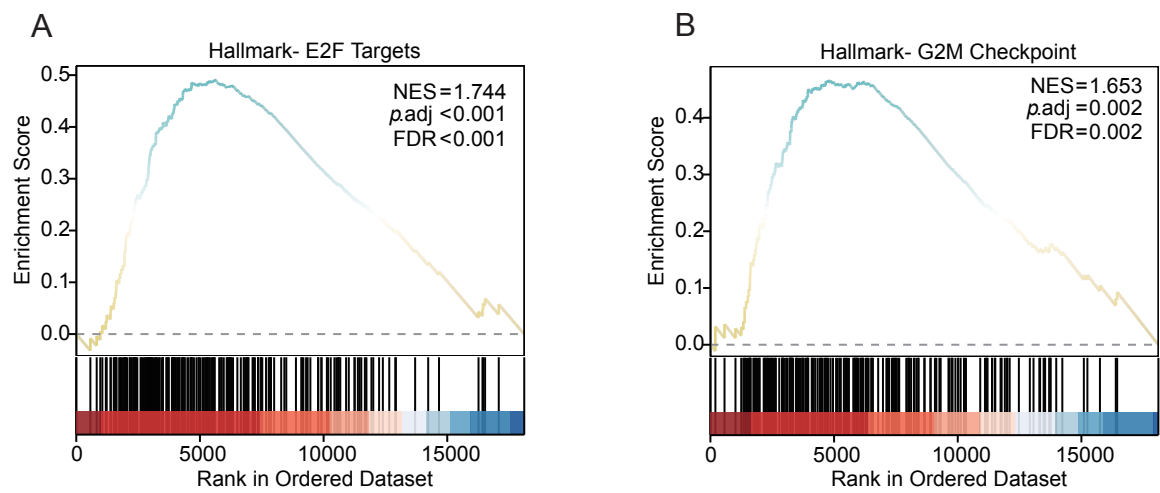

Supplement: S8 Fig — (PDF) [file pone.0349410.s008.pdf]
